# A Comprehensive Mechanical Examination of ABS and ABS-like Polymers Additively Manufactured by Material Extrusion and Vat Photopolymerization Processes

**DOI:** 10.3390/polym15214197

**Published:** 2023-10-24

**Authors:** Zorana Golubović, Ivan Danilov, Božica Bojović, Ljubiša Petrov, Aleksandar Sedmak, Žarko Mišković, Nenad Mitrović

**Affiliations:** 1 University of Belgrade, Faculty of Mechanical Engineering, 11120 Belgrade, Serbia; 2 Tipteh d.o.o., 11120 Belgrade, Serbia; 3 Innovation Center of the Faculty of Mechanical Engineering, 11120 Belgrade, Serbia

**Keywords:** additive manufacturing, FDM, SLA, DLP, ABS filament, ABS resin, mechanical properties, microscopy, hardness, 3D scanning

## Abstract

Additive manufacturing technologies have developed rapidly in recent decades, pushing the limits of known manufacturing processes. The need to study the properties of the different materials used for these processes comprehensively and in detail has become a primary goal in order to get the best out of the manufacturing itself. The widely used thermoplastic polymer material acrylonitrile butadiene styrene (ABS) was selected in the form of both filaments and ABS-like resins to investigate and compare the mechanical properties through a series of different tests. ABS-like resin material is commercially available, but it is not a sufficiently mechanically studied form of the material, which leads to the rather limited literature. Considering that ABS resin is a declared material that behaves like the ABS filament but in a different form, the objective of this study was to compare these two commercially available materials printed with three different 3D printers, namely Fused Deposition Modelling (FDM), Stereolithography (SLA) and Digital Light Processing (DLP). A total of 45 test specimens with geometries and test protocols conforming to the relevant standards were subjected to a series of tensile, three-point bending and compression tests to determine their mechanical properties. Characterization also included evaluation of morphology with 2D and 3D microscopy, dimensional accuracy of 3D scans, and Shore A hardness of each material and 3D printing process. Tensile testing results have shown that FDM toughness is 40% of the value for DLP. FDM elongation at break is 37% of DLP, while ultimate tensile stress for SLA is 27% higher than FDM value. Elastic modulus for FDM and SLA coincide. Flexure testing results indicate that value of DLP flexural modulus is 54% of the FDM value. SLA strain value is 59% of FDM, and DLP ultimate flexure stress is 77% of the value for FDM. Compression test results imply that FDM specimens absorb at least twice as much energy as vat polymerized specimens. Strain at break for SLA is 72% and strain at ultimate stress is 60% of FDM values. FDM yield stress is 32% higher than DLP value. SLA ultimate compressive stress is half of FDM, while value for DLP compressive modulus is 69% of the FDM value. The results obtained are beneficial and give a more comprehensive picture of the behavior of the ABS polymers used in different forms and different AM processes.

## 1. Introduction

Additive manufacturing (AM), or 3D printing, is a widely used technology in various prototyping processes and in the production of complex shapes and geometries. With the rapid improvement in the manufacturing of final products, the production and development prospects in industry and scientific research have changed greatly. Unlike conventional machining methods, i.e., material subtraction, molding, or casting, which leave behind a lot of waste material, AM technologies have revolutionized the production of final parts by applying one layer at a time and consuming less material. Today, it is possible to quickly manufacture various parts and objects directly from computer-designed drawing data. The manufacturing process with product development and production cycle is significantly shortened by 3D printing, while at the same time the usability of materials is greatly improved by this production approach. AM enables customization of parts with geometries considered difficult for conventional processes, which is another advantage for producing versatile, customized parts and leads to simplification of design, logistics, and maintenance [1]. Due to the diversity of materials that can be printed (polymers, metals, ceramics, composites, biological materials), a wide range of possibilities can be realized with these technologies, allowing access to a variety of different fields (automotive, aerospace, architecture, biomedical, applied sciences, education, etc.) [2,3]. To date, there are four different groups of additive manufacturing processes, namely photopolymerization, material extrusion, powder bed melting, and binder jetting [4,5]. The methodology of AM production is based on the creation of successive cross-sectional layers of an object. The AM process starts with the creation of a three-dimensional solid model, previously scanned or modelled in a digital file CAD. The model is then sliced into thousands of layers in appropriate software (Chitubox V1.9.0. for DLP/SLA and Simplify V5 for FDM), depending on the available resolution. Sequential layering using selective material deposition, polymerization process, or energy fusion of the raw material creates each layer.

Among the various AM technologies and materials, extrusion-based fused deposition modeling (FDM), vat polymerization stereolithography (SLA), and digital light processing (DLP) were selected for this research. Different studies dealt with adjusting the material properties and examination of its anisotropy with regard to the mechanical or fracture behavior [6,7]. As a material extrusion process, FDM is a widely used AM technology for plastic part manufacturing, and most of the literature focuses on the mechanical properties of FDM parts. One of the disadvantages of FDM is the poor surface accuracy due to the filament voids from nozzle diameter. In the case of SLA, situation is different because of the smaller laser beam and different layering of the material [8]. FDM is a process in which thermoplastic filament materials are first melted and then extruded layer by layer on the hot build platform while forming a specific shape [9]. SLA is the vat photopolymerization technology first developed and extensively studied to improve its process performance [10]. It is a process in which photosensitive liquid resin is cured layer by layer by a laser beam with UV light [11]. Although it belongs to the same technology of photopolymerization, unlike SLA, DLP exposes the layers with an image to cure the desired voxels in a 2D plane simultaneously [12].

In this study, FDM, SLA, and DPL printers were used to produce standardized specimens for mechanical testing. FDM, SLA, and DLP 3D printing processes have been studied separately and comparatively in terms of the processes themselves and the properties of the manufactured parts and their behavior [13,14,15,16,17,18]. As the capabilities and applications of 3D printing become more diverse, the study of different materials and their properties continues to increase and expand. Polymers are of particular interest, leading to the development of different blends and types to achieve the desired mechanical properties of the printed parts. Various polymeric materials, such as acrylonitrile-butadiene-styrene (ABS), polylactic acid (PLA), nylon, and polycarbonate (PC), are used vastly for FDM printing processes [19]. ABS material in particular, and properties of ABS parts printed using FDM processes, have been extensively studied [20,21,22,23]. ABS has been shown to be an excellent thermoplastic amorphous polymer with good mechanical and excellent thermal properties. ABS filament material has been extensively studied for FDM 3D printing processes. It has high stress and strain values, good electrical properties, chemical resistance and processability, and dimensional stability, but it emits an unpleasant odor during printing [24,25]. Garg et al. investigated the effect of part orientation along the X, Y, and Z axes and with four different print orientations on surface roughness, tensile strength, flexural strength, and wear, confirming the conclusions, that the mechanical strength and surface roughness of FDM specimens are highly dependent on the part orientation and that different screen angles together with different part orientations exhibit highly anisotropic behavior [26]. Similar conclusion is drawn from another study that the mechanical properties of the specimens can differ to a large extent when the orientation of the specimen is changed during the printing process [27]. Various finite element models have been developed to simulate the process of ABS 3D printing and to facilitate parameter selection [28,29]. Specific properties need to be known, relationships between materials properties, cyclic limits, mechanical testing to assess whether a particular additively manufactured part can meet the requirements for its application [30].

With intention to draw the parallel and compare mechanical behavior and morphology of different forms of ABS materials, in this research is, aside the ABS filament printed on FDM, used ABS-like resin for specimen preparation on SLA and DLP printers. The resins used for SLA and DLP processes are photosensitive thermoset polymers, i.e., polymers that are in soft solid or viscous liquid form and reversibly polymerized (cured) [17]. ABS-like resins can be used for 3D printing of parts with moderate detail, high strength, and satisfactory functionality [31]. Their properties influence the optical, chemical, and mechanical properties of the final product [32]. ABS-like resin consists of three parts, acrylonitrile, butadiene and styrene, which are further forming a two-phase system, i.e., styrene-acrylonitrile copolymer is forming the SAN matrix and the polybutadiene rubber particles are in the dispersed phase. These two phases are bonded to the matrix SAN layer and, in this way, are polymer compatible. Each of these components and their ratio affects the specific properties of the resin [33]. Thus, the acrylonitrile affects the heat and chemical resistance and surface hardness of the final product, the butadiene affects toughness and impact strength, and the styrene affects processability, stiffness, and strength [34].

The applications of these resins in industry are diverse and include computer consoles, household materials, interior and exterior parts of automobiles, luggage, and various pipes [35]. One of the studies shows that ABS-like resins have a tensile strength of 39–60 MPa, while their elastic modulus varies between 0.7 and 2.2 GPa. However, it is important to emphasize that the manufacturing strategy, processing parameters, and even testing conditions play a significant role in the production steps and final results [36].

Billing et al. examined standard LCD UV-curing photopolymer rapid resin and an ABS-like LCD UV-curing photopolymer rapid resin, together with manufactured nanoparticle-reinforced photocurable resin-based nanocomposites. It was shown that factory ABS-like resin outperformed other tested materials leading to the increase of 24.7% in tested abrasion resistance. Authors concluded that this characteristics makes the ABS-like resin adequate for applications where low stresses, but high traffic are present [37].

The influence of print orientation has not yet been adequately studied, with general characteristics taking precedence over mechanical properties [38]. In a previous work, a printing orientation of 45° was investigated under the same conditions, resulting in higher values of fracture stress, elastic modulus and maximum strain for 3D printing SLA, and lower values in the case of DLP [39]. Post-processing, i.e., immersion in acetone solution, also played an important role in improving the mechanical properties, resulting in increased ductility, lower ultimate load, and tensile strength of the printed ABS specimens [19].

ABS-like resin material is commercially available, but it is not a sufficiently mechanically studied form of the material, which leads to the rather limited literature. It is well known that each manufacturer has its own formulations of the resin materials and, in the case of ABS, they are often made to match the filament in some of the properties. For this reason, ABS materials from the same manufacturer, for both the filament and the resin, are chosen for this research.

To the knowledge of the authors of this manuscript, no similar studies have been conducted comparing filaments and resin made from the so-called “same” material, in this case ABS. ABS filaments have been thoroughly investigated in the context of various mechanical tests, but this is not the case for ABS-like resin. Numerous factors affect the quality of the finished part, and therefore it is necessary to continuously test materials in various 3D printing processes. Factors to consider include raw material characteristics, printer conditions, environmental conditions, etc. Even with the same printer, print quality can vary from batch to batch. For this reason, various investigations are crucial to obtain a more comprehensive picture of the possible influences, resulting in parts whose quality meets the required standards.

Bearing in mind that ABS resin is declared material acting like the ABS filament, but in different form, the intention of this study was to compare these two commercially available materials, printed with three different printers, i.e., FDM, SLA and DLP, according to the data taken from the manufacturer’s website. Compared properties included the mechanical characteristics of tensile, compression and flexure testing, morphology evaluation with 2D and 3D microscopy, 3D scanned dimensional accuracy, and Shore A hardness of each material and 3D printing process.

## 2. Methodology

### 2.1. Specimen Preparation

Two commercially available materials were used in this research, ABS filament (Creality, Shenzhen, China) and ABS-like resin (Creality, Shenzhen, China). All specimens underwent complete set of testing planned within this study.

Three different geometries, 45 specimens in total (Table 1), were modelled in dedicated CAD software (SolidWorks 2020, Dassault Systèmes SE, Vélizy-Villacoublay, France), according to the corresponding standards, and used for the mechanical tests and characterizations carried out within the scope of this research.

**Figure 1:**
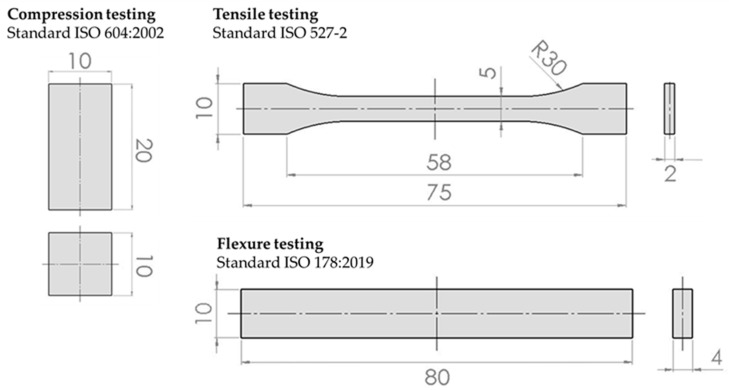
Specimen dimensions according to the standards [40,41,42].

Specimen’s geometries are in compliance with the specified standards, i.e., ISO 527-2 standard for tensile testing [40], ISO 604:2002 for compression testing [41], and ISO 178:2019 for flexure testing [42] (Figure 1).

FDM printer utilized is Creality CR-10 smart pro FDM (Creality, Shenzhen, China), SLA is Kings 600 Pro, Shenzhen, China and DLP is Creality LD-002R, Shenzhen, China. It should be mentioned that SLA is an industrial 3D printer and DLP is common desktop 3D printer. Printing parameters are given in Table 2. Infill density of all specimens was 100%, with grid infill pattern and with 90° print orientation. After printing, all the specimens were stored and tested at room temperature of 23 °C and humidity of 55% RH.

Final quality of the part is dictated by the layer thicknesses, which are different in the case of material extrusion and vat photopolymerization processes, because of their printing resolutions [43]. For FDM, a layer thickness was 0.24 mm, while for SLA and DLP, it was 0.05 mm.

### 2.2. Material Examination

#### 2.2.1. 3D Scanning

After 3D printing is finished on each printer, the specimens were measured and scanned in order to compare original digital models with printed specimens, and to determine the dimensional accuracies and deviations. For the purpose of scanning the geometric dimensions, an Atos Core 200 (GOM, Braunschweig, Germany) non-contact 3D optical scanner is used, and for data acquisition and processing, GOM Inspect 2020 software is utilized. All specimens were sprayed before scanning, to obtain better surface detection by the scanner. Scanned spatial images are given, and the volume of the specimens could be obtained, which would not be possible with manual measurements (calipers), because of the changes and irregularities of shape which occurred during printing [44].

#### 2.2.2. Mechanical Testing

Mechanical testing was carried out on the universal testing machine Shimadzu AGS-X (Shimadzu Corp., Kyoto, Japan) equipped with load cell of 100 kN capacity. According to standards, the speed of testing was 1 mm/min. The average stress–strain curves for five specimens per each material, and 3D printing processes that undertake the each of three mechanical tests, are computed in Matlab R2022b software. Anisotropic behavior of materials through different printing processes, and understanding of the tensile, compressive, and flexural properties of printed parts is crucial for complete property characterization [45].

#### 2.2.3. Hardness

In order to measure Shore A hardness values, commercial measurement device SAUTER HDA100-1 (Conrad, Berlin, Germany) was used. According to the proposition of the ASTM D2240 standard [46], 5 indentations from different places were taken for each surface. Hardness measurements are a significant part of material characterization in order to determine the ability of material to resist and recover from mechanical indentations or abrasions [10,47]. Different properties of the material influence the hardness; for example, the water-absorbing property of the ABS-resins reduces the hardness of the 3D-printed parts when it increases [48].

#### 2.2.4. Microscopy

Optical microscopy aimed to evaluate the internal structure of the material at the cracked places after the mechanical testing. Micrographs were obtained using a laboratory-grade 3D Digital Video Microscope KH-7700 (Hirox, Tokyo, Japan), together with a the Mustool G600 Digital Portable 2D Microscope (Shenzhen, China). Depending on the specimen and cracking, magnification range was between 50 and 100×. Optical microscopy can give significant insight in the surface morphology, material layering, air effects, and fusion of the filaments, as well as the surface finish and thicknesses [49]. All the parameters lead to better understanding of different modifications that occur during printing that affect mechanical behavior, and possible ways to improve the 3D printing processes and post-processing [50].

## 3. Results

In this study, the mechanical performance of 45 specimens made of ABS filament and ABS-like resins are obtained to ratify the printed specimen’s behavior for particular applications. Additionally, the experimental conclusions based on geometry scans, hardness testing, and fractured surface microscopy are presented as well.

### 3.1. Mechanical Testing 

**Figure 2:**
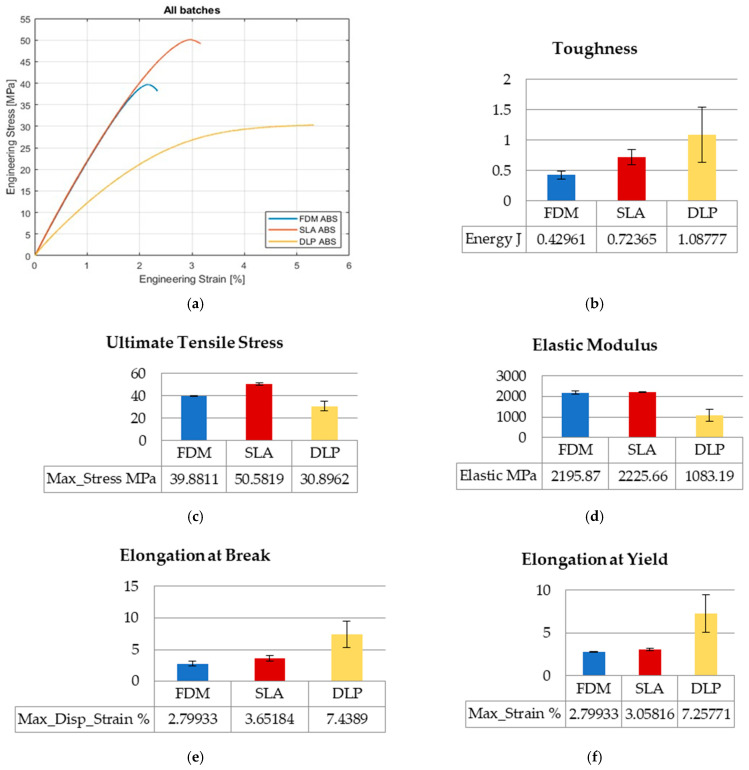
Tensile testing results: (**a**) stress–strain curves; (**b**) toughness; (**c**) ultimate tensile stress; (**d**) elastic modulus; (**e**) elongation at break; (**f**) elongation at yield.

**Figure 3:**
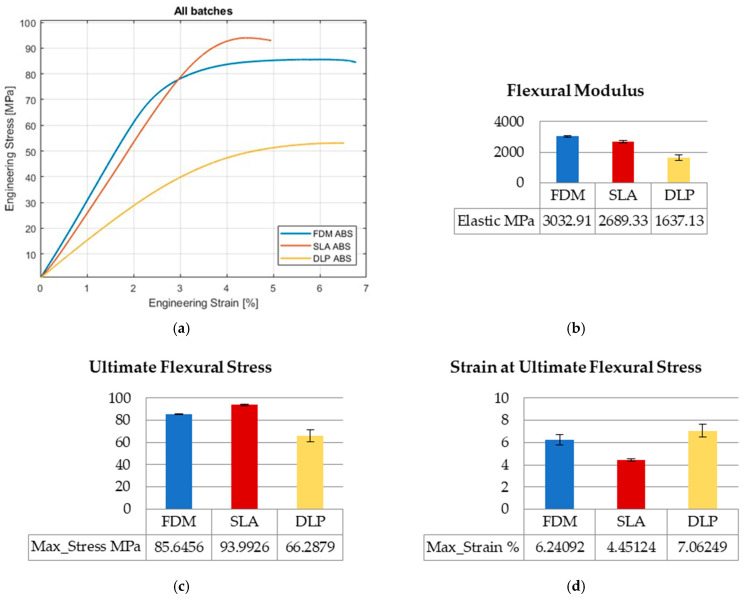
Flexure testing results: (**a**) stress–strain curves; (**b**) flexural modulus; (**c**) ultimate flexural stress; (**d**) strain at ultimate flexural stress.

**Figure 4:**
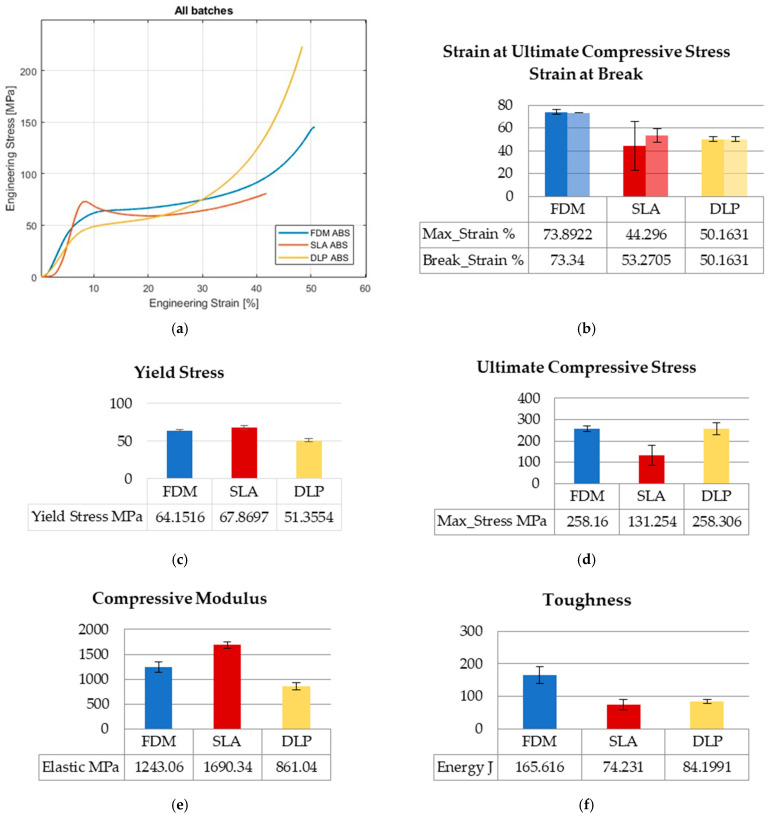
Compression testing results: (**a**) stress–strain curves; (**b**) strain at ultimate compressive stress–strain at break; (**c**) yield stress; (**d**) ultimate compressive stress; (**e**) compressive modulus; (**f**) toughness.

Values of engineering stresses are averaged by computation until the first of five specimens reaches break point. In that calculating manner, the distinctive results considering toughness and ultimate values could be revealed in graphs regarding the overall collected data range. The graphs show average values of mechanical properties along with the standard errors. The curves of the three AM technologies and the mechanical properties compared in graphs are presented in Figure 2, Figure 3 and Figure 4 for tensile, flexure, and compression testing results.

#### 3.1.1. Tensile Testing

Tensile tests were performed for five specimens per printing technology, totaling fifteen overall. Stress–strain curves for FDM and SLA specimens exhibit similar behavior, while DLP-printed ABS specimens behave differently (Figure 2a). DLP specimens appear to be more ductile than FDM and SLA, which is confirmed with the toughness, elongation at break, and elongation at yield values. The value of FDM toughness is 40% that of DLP toughness, and SLA toughness is 67% that of DLP toughness (Figure 2b). The elongation at break value for FDM is 37% of DLP and, for SLA, is 50% of DLP elongation’s value (Figure 2e). The elongation at yield value for FDM is 38% of DLP and, for SLA, is 42% of DLP elongation’s value (Figure 2f). The elastic modulus for FDM and SLA have close values in the range of 0.1% (Figure 2d) and stress–strain curves coincident with the same slope in Figure 2a. The elastic modulus value for DLP is almost half of SLA elastic modulus value (Figure 2d). The highest value for ultimate tensile stress occurs for SLA technology, as it is 27% higher than FDM value and 64% higher than DLP value (Figure 2c). Opposed to the FDM and SLA results, the repeatability of the mechanical properties of DLP-printed specimens is not so representative.

#### 3.1.2. Flexural Testing

Flexural tests (three-point bending tests) were performed for five specimens per printing technology, totaling fifteen overall. Stress–strain curves for FDM-, SLA-, and DLP-printed ABS specimens (Figure 3a) present different behaviors and have different slopes, because the flexural modulus has different values. DLP has the lowest flexural modulus value, which is 54% of FDM value and 60% of SLA value (Figure 3b). FDM and DLP specimens behave in a more ductile manner, and endure similar strain by the ultimate flexural stress strains values, which is opposite to SLA. That is confirmed in Figure 4d; the SLA value is 59% of FDM, and 40% of the DLP strain value. The highest value for the ultimate flexure stress occurs for SLA technology, i.e., 9.7% higher than FDM, and 42% higher than DLP (Figure 3c). Compared to FDM and SLA results, the range of mechanical properties of DLP-printed specimens is wider.

#### 3.1.3. Compression Testing

Compression tests were performed, as in previous tests, for five specimens per printing technology, totaling fifteen overall. Stress–strain curves for FDM-, SLA-, and DLP-printed ABS specimens (Figure 4a) display quite different behaviors among printing processes. SLA yield stress (Figure 4c) is close to FDM, and 32% higher than the DLP value. The compressive modulus (Figure 4e) is 36% higher than FDM, and almost double the DLP value. A similar value for ultimate compressive stress occurs for FDM and DLP technology (Figure 4d), although the curves in Figure 4a are not going along with that statement. The computation averages the values of engineering stress until first of five specimens reach break point, as is said before, which causes that distinction in ultimate values regarding range of overall collected data. FDM specimens show dominant behavior considering the values for strain at break and strain at ultimate stress (Figure 4b). DLP has the same values for strain at break and strain at ultimate stress, which amount to 68% of FDM strains. The SLA strain at break value is 72% of the FDM value, and the strain at the ultimate stress value is 60% of the FDM value. Additionally, FDM specimens absorb at least twice as much energy as vat polymerized specimens, which can be seen in Figure 4f.

### 3.2. Microscopy

**Figure 5:**
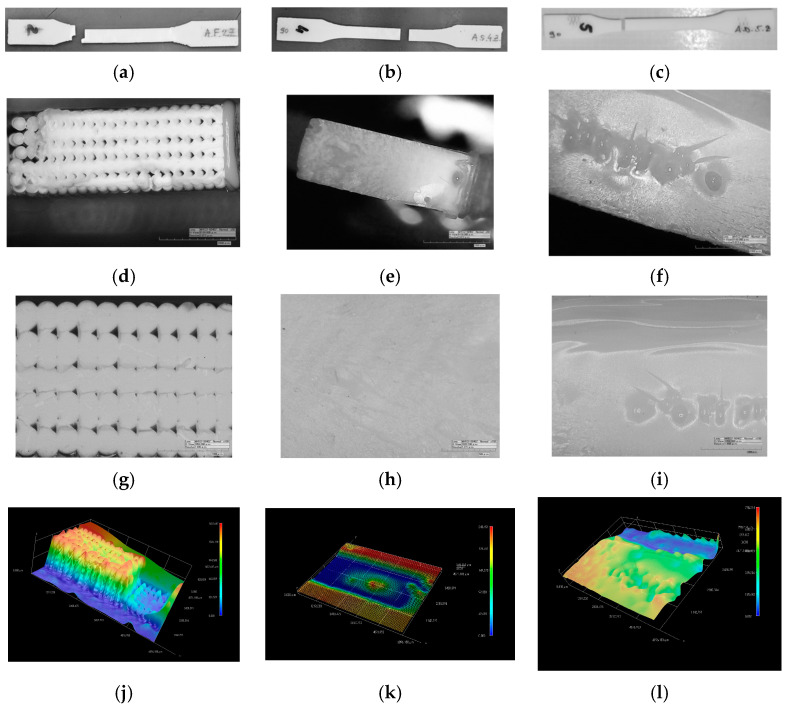
Tensile testing fractured surface images: (**a**) FDM; (**b**) SLA; (**c**) DLP. Two-dimensional microscopy of fractured surface (**d**,**g**) FDM; (**e**,**h**) SLA; (**f**,**i**) DLP. Three-dimensional microscopy of fractured surface (**j**) FDM; (**k**) SLA; (**l**) DLP.

**Figure 6:**
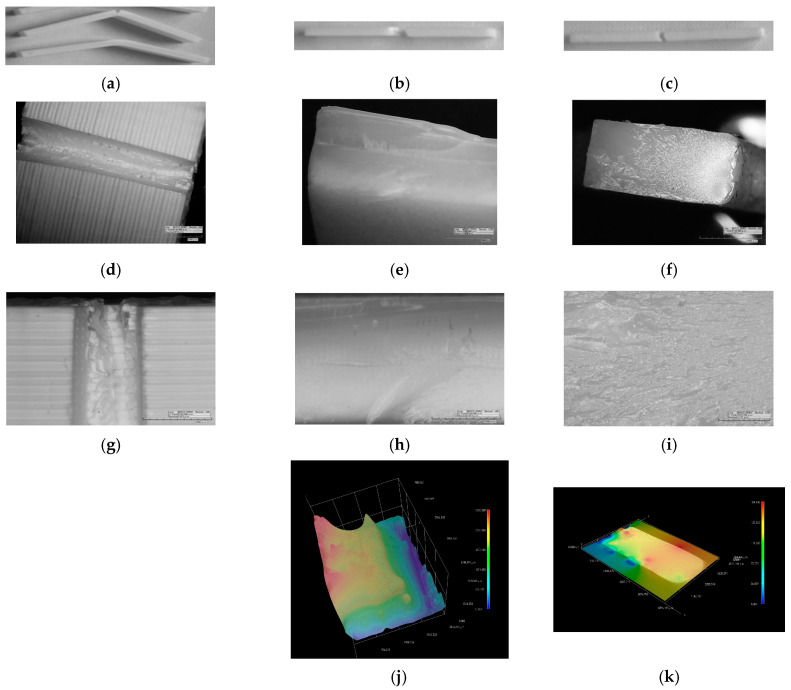
Flexural testing fractured surface images: (**a**) FDM; (**b**) SLA; (**c**) DLP. Two-dimensional microscopy of fractured surface (**d**,**g**) FDM; (**e**,**h**) SLA; (**f**,**i**) DLP. Three-dimensional microscopy of fractured surface (**j**) SLA; (**k**) DLP.

**Figure 7:**
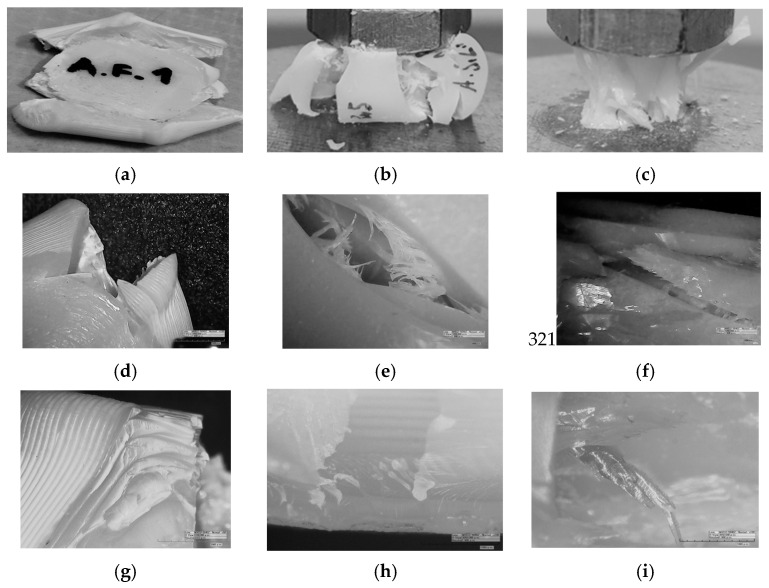
Compression test fractured surface images: (**a**) FDM; (**b**) SLA; (**c**) DLP. Two-dimensional microscopy of fractured surface (**d**,**g**) FDM; (**e**,**h**) SLA; (**f**,**i**) DLP.

After mechanical testing was finished, fractured specimens are observed with 2D and 3D microscopy to obtain better insight into the morphology of different prints and materials. Micrographs of all three AM technologies are presented in Figure 5, Figure 6 and Figure 7, i.e., tensile, flexure, and compression fractured specimens, respectively.

#### 3.2.1. Tensile Testing

In Figure 5b,c, the fractured places in the narrow section of specimens can be observed in case of vat photo-polymerized specimens, and fracture lines are clear and straight. During tensile testing, FDM specimens broke at necking position (Figure 5a), and some of them have stair-like fracture line. The images in Figure 5d–f are fractured surfaces gathered at magnification 50×, and indicate different fracture modes for specimens made by different AM technologies. Extruded melted ABS filaments, built in layers, are stair-like broken and demonstrate fractures due to extending of filaments (Figure 5d). In Figure 5g, the bigger or smaller gaps between filaments are observed and filament cross-section is in shape of deformed circle. At the 3D micrograph in Figure 5j, the crescent (ductile) mode at the ridge is present, and the trough mode at upper and lower stair-like fracture appears as a consequence of high-order fretting. The brittle mode of fractured surface for SLA-printed specimens is observed in Figure 5e, and irregularities in shape of bubbles as well. The outer layers of the cross-section exhibit shear in the corners at the left side of micrographs. Figure 5h shows homogenous material structure for SLA building technology and striation. The size of bubble visible in 3D image (Figure 5k) is around 100 μm. Fractured surface of DLP specimens has irregularities in shape of bubbles in the middle of the micrograph in Figure 5f. A zoomed view of the bubble cluster is presented in Figure 5i. The 3D image in Figure 5k indicates the size of bubbles, which are up to 240 μm.

#### 3.2.2. Flexure Testing

In Figure 6a, it is shown that three-point bending does not cause fracture for all FDM specimens. Two of the five specimens are bent but remain unbroken, and three broke, but did not separate in two pieces (representative specimens for both cases are shown in Figure 6a). Bent fracture lines in the middle of broken vat photo-polymerized specimens can be observed in Figure 6b,c. Micrographs in Figure 6d–f present fractured surfaces gathered at magnification 50×, and indicate different fracture mode for specimens made by using different AM technologies. ABS filaments built in layers are broken in a cut-off manner (Figure 6d). Only a few layers stay unbroken from the load pin side, and demonstrate the ductile-like behavior of filaments (Figure 6g). In case of FDM built specimens, the 3D image capture was unsuccessful. Figure 6e confirms the brittle mode of the fractured surface for SLA-printed specimens, along with ridges that protrude. This ridge is part of complementary half of the broken specimen. More details of uneven fractured surfaces are observed in Figure 6h. The 3D image of the SLA fractured surface (Figure 6j) shows a ridge from one side and a valley from opposite side. The fractured surface of DLP-printed specimen differentiates two zones: (1) ductile zone with striation and (2) the brittle zone in the upper left corner of 2D image in Figure 6f. The highly developed striation could be observed closer in Figure 6i, while in Figure 6k, the flat fractured surface in 3D image is present.

#### 3.2.3. Compression Testing

In Figure 7a, it is shown that compression of FDM specimens first lead to barreling, followed with squashing and aside exploding of the central part, which was caused by the release of tension from compressed air enclosed by the layers. SLA specimens undergo barreling during compression and crushing in bloom-like form. For some specimens, spreading and widening occurs in single side of prism, and for others, for two or even four sides, like for this representative specimen in Figure 7b. After the barreling, the DLP specimen bursts (representative in Figure 7c) and splinters separate from it uncontrollably. Micrographs in Figure 7d–f are separations of broken specimen gathered at magnification of 50×, and indicate different fracture modes for specimens made by using different AM technologies. The ABS filament is stacked tightly in layers and broken in the corner (Figure 7d,g), and from the broken corner, where the filaments are torn out, the ductile behavior of filaments can be noticed. The SLA specimens exhibit brittle fractures in outer layer (Figure 7e) and torn thin fibers. Figure 7h shows, in detail, a view inside of crack exposed layers in broken side of specimen. The sharp splinters in Figure 7f confirm the brittle mode that the DLP specimen undertakes. Splinters are monolith spear-like or tiny spikes, which are observed in Figure 7i altogether with layers. In the case of compression, 3D micrographs were not significantly successful.

### 3.3. 3D Scanning

**Figure 8:**
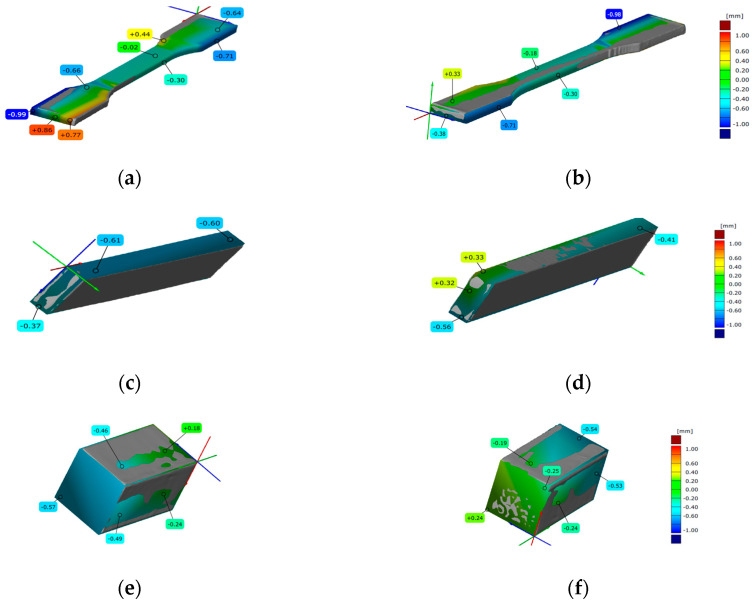
Three-dimensional scanned geometry accuracy of FDM-printed specimens for: (**a**,**b**) tension testing; (**c**,**d**) flexure testing; (**e**,**f**) compression testing.

**Figure 9:**
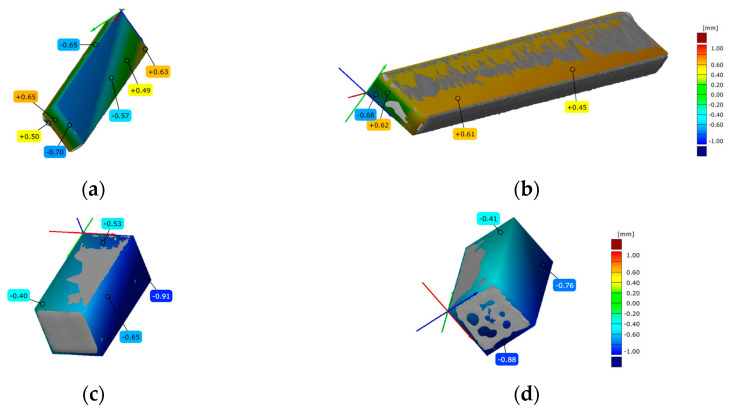
Three-dimensional scanned geometry accuracy of SLA-printed specimens for: (**a**,**b**) flexure testing; (**c**,**d**) compression testing.

**Figure 10:**
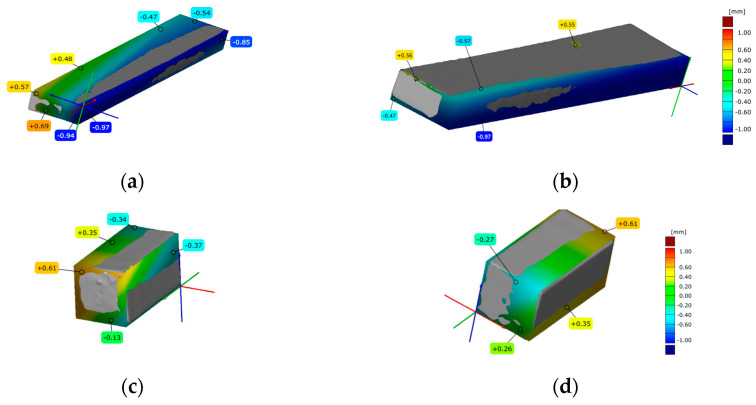
Three-dimensional scanned geometry accuracy of DLP-printed specimens for: (**a**,**b**) flexure testing; (**c**,**d**) compression testing.

Before each mechanical testing, among the printed specimens representative specimen was scanned. Scanned specimens, after overlapping with the digital model, for all three types of FDM, SLA and DLP AM processes are presented in Figure 8, Figure 9 and Figure 10, for all three types of mechanical testing.

#### 3.3.1. FDM Scanned Specimens

In Figure 8, both sides of the scanned FDM specimens are compared to appropriate CAD models. A representative specimen for tensile testing is shown in Figure 8a,b. The positions of detected deviations and the values (from −0.99 to +0.86) are tagged onto scanned specimen. The positions and the values (from −0.61 to +0.33) of deviations are marked onto scanned representative specimen for flexure testing in Figure 8c,d. Figure 8e,f shows representative specimens for compression testing, along with the deviations values (from −0.57 to +0.24) and their sites. Graduated bars indicate the error range.

#### 3.3.2. SLA Scanned Specimens

In Figure 9, both sides of the scanned SLA specimens are compared to appropriate CAD models. The CAD model and the scan of the representative SLA specimen for tensile testing could not be overlapped. In Figure 9a,b, the positions of detected deviations and the values (from −0.70 to +0.65) are marked onto scanned specimen for flexure testing. The deviations’ values (from −0.91 to −0.40) are set onto a scanned representative specimen for compression testing (see Figure 10c,d). Graduated bars indicate the error range.

#### 3.3.3. DLP Scanned Specimens

Overlapping of the representative DLP specimen scan model and CAD model for tensile testing was not successful. In Figure 10, both sides of the scanned DLP specimens for flexure and compression testing are compared to suitable CAD models. The deviations’ values (from −0.97 to +0.69) are set onto a scanned representative specimen for flexural testing (Figure 10a,b). Figure 10c,d shows of detected deviations positions and the values (from −0.61 to +0.27) are marked onto scanned representative specimen for compression testing.

### 3.4. Hardness

The conventional test covered by the ASTM D2240 standard for elastomers was performed five times per each type of printed ABS specimens of both material forms. The calculated average values of hardness for ABS material printed using FDM, SLA, and DLP technology are 90A, 87A, and 85A, respectively. Therefore, filament and resin printed specimens show distinctive hardness, regardless of the 3D printing technology. Specimens made from ABS filament are considered hard elastomers. Otherwise, specimens made of ABS resin are considered to be medium to hard elastomers.

## 4. Discussion

There are several factors that limit the use of AM technology in manufacturing, such as material cost, machine cost, the speed of the printing process, repeatability, reproducibility, and special characteristics of the finished parts [51]. The quality of 3D-printed specimens can be evaluated using various methods with respect to the assessment point and possible planned improvements. Various scanners, micrometers, calipers, and scales are used to obtain the data by measuring the morphology, dimensions, geometry deviations, surface roughness, volume density, weight, and other parameters of the finished parts. In this research, different methods were used for characterization and quality control of the finished specimens, e.g., 2D and 3D microscopy, hardness device, and 3D scanner.

Experimental results and a comparison of the ABS specimen’s mechanical behavior between three different AM processes show ductile or brittle behavior for filaments and resin, respectively. Considering the tensile mechanical performances, the vat polymerization processes printed parts that have better performances than FDM ones. Particularly, SLA parts are stronger, while DLP parts are tougher. For the flexural performance, specimens printed with FDM process achieve the highest flexural modulus, which generates the flexible and unbreakable parts. SLA process provide the highest value for ultimate flexure stress, thus strong parts are produced, while DLP process reaches the highest strain which occurs at flexural stress, and with that tougher parts are made. Results from compressive tests show that parts are tougher, and SLA part are behave in stiffer manner. DLP and FDM part endure similar ultimate stress.

Presented results could be considered as a suggestion for adequate AM process selection for certain engineering applications of ABS 3D-printed parts.

Two-dimensional and 3D microscopy was used to observe the morphology of the fractured specimens and patterns for mechanically tested specimens. Micrographs after tensile testing, reveal ductile fracture for FDM, and brittle fracture along with bubbles defect for vat polymerized specimens. After three-point bending testing, FDM specimens remained in one piece, either unbroken, or broken but attached. Bending of vat polymerized specimens led to the brittle mode of uneven fractured surface with developed striation. Compression of specimens starts with barreling. At the end, FDM specimens have the crack growth in the squashed central part and one or more sides exploded, caused by releasing the tension of compressed air enclosed by layers. SLA specimens ended in a bloom-like crashing form. DLP specimens burst at the end, along with sharp splinters separating from the core. Generally, FDM specimens have ductile behavior and vat polymerized specimens have brittle behavior during compression.

When evaluating print quality, ensuring dimensional repeatability and reproducibility of printed parts is the next important step [52]. While repeatability ensures that quality remains consistent across samples, reproducibility means that the same results and accuracies can be achieved across different 3D printing processes and is an important factor in volume production. Reproducibility depends on the type of AM technology, 3D printers, materials, production process, and post-processing [53].

The specimens from this research were 3D scanned immediately after 3D printing to compare geometry accuracy, or possible deviations with the CAD model. Of all the scans, the most interesting are presented to provide insight into the overlaps. There are differences due to surface finish and impregnation of the spray used for better visualization. Also, parts of the scanned 3D models were not complete, and in some places, smaller parts of the measurement volume were missing.

Standardized specimen geometries for this research were: (1) dog-bone for tensile testing, (2) bar for flexure testing, and (3) brick for compression testing. The specimens’ 3D scanned models and their comparison to appropriate CAD models point out the AM technology accuracy. Scans have shown that FDM is a more suitable AM technology for printing long and thin parts, since vat polymerized tensile specimens were warped. Polymerization technologies exhibit worse accuracy compared to FDM in case of long bars, since the range of deviation is the smallest for FDM. In the case of thick and short printed specimens, SLA obtained the most accurate printing.

Generally, vat polymerization technology is better for fine detail printing, and leaves a finer surface finish. Otherwise, additional post-processing, such as polishing, is required to remove material from surfaces that had attached support.

ABS resins have lower Shore *hardness* compared to the filament and, therefore, are more flexible, making DLP-printed ABS resin suitable for applications that require bending or stretching. Opposed to it, FDM-printed ABS filament, as a harder material, may withstand stresses (or pressures).

## 5. Conclusions

A comprehensive understanding of the properties of various polymer materials still remains partial. With the goal of better understanding the mechanical behavior of ABS as a resin material, standardized specimens were prepared using two extrusion and vat AM technologies for all three types of mechanical testing. This study extends the knowledge of mechanical behavior and properties based on an experimental investigation and comparison between two commonly used AM technologies—fused filament extrusion (FDM) and vat photopolymerization (SLA and DLP). The focus was to determine the parallels between ABS filament and ABS-like resin material and their differences.

FDM printing process have shown satisfactory results and confirmed previously known findings. The differences in the mechanical properties of the ABS resin in relation to the printing technology are in favor of the SLA printing technology in terms of the curing process of the resin, which is periodically exposed to UV laser light and consequently builds up a fine and dense structure, compared to DLP technology, where whole layers are flashed at once to cure the resin in the resin tank. It can be concluded that the printing technology has a predominant influence on the mechanical properties.

In addition to providing interesting and useful characterization data for ABS materials, the results of this study shed light on possible material selection with respect to the required applications, keeping in mind that scientific information on ABS-like resins is still limited.

## Figures and Tables

**Table 1:** Specimen distribution by material and process.

Material	3D Printing Process	Tensile	Flexure	Compression	∑
ABS	FDM	5	5	5	15
ABS-like	DLP	5	5	5	15
	SLA	5	5	5	15
					45

**Table 2:** Printing parameters for all the processes and specimens.

Description	FDM	Description	SLA	Description	DLP
Material	ABS filament	Material	ABS resin	Material	ABS resin
Layer thickness	0.24 mm	Layer thickness	0.05 mm	Layer thickness	0.05 mm
Nozzle diameter	0.4 mm	Laser beam size	0.08 mm	Bottom layer count	10
Filament diameter	1.75 mm	Scanning speed	1.5 m/s	Exposure time	8 s
Printing temperature	250 °C	Wavelength	355 nm	Wavelength	405 nm
Build platform temperature	90 °C			Bottom exposure time	80 s
Printing speed	60 mm/s			Bottom lift speed	100 s

## Data Availability

Data can be requested via the corresponding author.
